# Relationship between Paraoxonase-1 and Arylesterase Enzyme Activities and SYNTAX I and II Scores in Patients with ST-Elevation Myocardial Infarction

**Published:** 2019-10

**Authors:** Erdoğan Sökmen, Mustafa Çelik, Serkan Sivri, Kenan Güçlü

**Affiliations:** 1 *Department of Cardiology, Ahi Evran University Training and Research Hospital, Kirsehir, Turkey.*; 2 *Department of Biochemistry, Ahi Evran University Training and Research Hospital, Kirsehir, Turkey.*

**Keywords:** *Myocardial infarction*, *ST elevation myocardial infarction*, *Clinical enzyme tests*, *Arylesterase*

## Abstract

**Background**: Although serum paraoxonase-1 (PON-1) and arylesterase (ARE) activities are linked to the presence of stable coronary arterial disease, their correlation with SYNTAX Score I (SS1) and SYNTAX Score II (SS2) has not been known well. Our aim was to determine the association between PON-1 and ARE activities, together with the genetic polymorphism of PON-1, and both SS1 and SS2 in patients with acute ST-segment elevation myocardial infarction (STEMI).

**Methods: **Consecutive patients with acute STEMI (n=102: 78 male, 24 female; mean age=61.14±12.25 y) admitted to the Emergency Department of Kırşehir Ahi Evran University Hospital between August 2018 and December 2018 were enrolled. PON-1 and ARE activities were determined on hospital admission. The SS1 and SS2 scores were calculated by using the angiographic and clinical data. Subsequently, the relationships between the activities of the enzymes, together with the genetic polymorphism of PON-1, and both SS1 and SS2 were interrogated.

**Results: **The mean SS1 and the mean SS2 were 19.8±9.7 and 32.3±11.5, respectively. The phenotype distributions of PON-1 were Q192Q (n=60), R192Q (n=35), and R192R (n=7). The respective PON-1 (U/L) and ARE (kU/L) activities were 514.85±29.34 and 216.82±36.72 in the low SS1 category; 527.60±56.31 and 203.95±55.97 in the intermediate SS1 category; and, 690.10±11.07 and 238.48±45.65 in the high SS1 category.PON-1 and ARE activities did not correlate with the SS1 categories, and varying SS2 scores. The distribution of the Q192R polymorphism was homogenous among the different SS1 and SS2 scores. The localization of acute STEMI also did not associate with the activities of either enzyme.

**Conclusion: **Admission serum PON-1 and ARE activities, together with the PON-1 Q192R genetic polymorphism, showed association neither with SS1 and SS2 nor with the localization of infarction in our acute STEMI patients.

## Introduction

Acute coronary syndrome still perpetuates its role as a significant contributor to morbidity and mortality the world over, despite advances both in interventional cardiology and medical therapy. Moreover, 1 out of 3 patients worldwide admitted to the hospital with acute myocardial infarction (MI) is liable to have acute ST-segment elevation myocardial infarction (STEMI).^1^^, ^^2^

During an episode of acute STEMI, there appears to be an increased burden of oxidative stress owing to an impairment in the antioxidant defense mechanism resulting from myocardial ischemia and reperfusion.^3^

High-density lipoproteins (HDLs) contribute to the preservation of normal vascular endothelial functioning through promoting endothelial cell integrity and homeostasis and constraining reactive oxygen species from disrupting cellular membranes and, thus, major endothelial functions.^4^ A previous study, on the other hand, suggested that HDLs underwent a number of structural and compositional modifications and, in turn, were rendered incapable of complementing these endothelial protective functions owing partly to a reduced paraoxonase-1 (PON-1) activity.^5^^-^^7^

PON-1 and arylesterase (ARE) are 2 HDL-associated and calcium-dependent isoenzymes. Encoded by the same gene, PON-1 and ARE are secreted by the liver into the bloodstream and possess similar active centers. Their main utility is to protect lipoproteins from lipid oxidation and, thereby, and provide defense against the generation of atherosclerosis.^8^^-^^10^ It was, accordingly, reported in the previous studies that the serum level of PON-1 was closely related to endovascular functioning and, hence, the extent of coronary arterial disease (CAD).^11^^, ^^12^ Although PON-1 displays a wide range of genetic polymorphism, which in turn culminates into the functional diversity of the enzyme, the same does not hold true for ARE. This genetic polymorphism of PON-1 was reported to stem mainly from the amino acid shift in the 192nd position of the molecule.^13^^-^^15^

The SYNTAX Score (SS1) was administered into the practice of invasive cardiology in an attempt to quantify better the complexity and extent of CAD,^16^^, ^^17^ wherein a greater SS1 implies a more extensive and complex status of the disease. A further SYNTAX scoring system (SYNTAX II Score [SS2]) was launched by Farooq et al.^18^ in an attempt to predict better the respective anticipated mortality following coronary artery bypass graft surgery and percutaneous coronary intervention (PCI). Unlike SS1, which comprises solely the variables of the coronary anatomy, SS2 encompasses both anatomical and clinical variables (2 anatomical and 6 clinical variables).

Although there are many studies suggesting that lower PON-1 and ARE activities are associated with the extent of CAD in patients with stable atherosclerotic cardiovascular diseases,^19^^-^^22^ the number of the studies investigating the association between PON-1 and ARE activities, together with the genetic polymorphism of PON-1, and SS1 in patients with acute STEMI is scant.^23^^, ^^24^

To our knowledge, there has been no study evaluating the correlation between PON1 and ARE activities and both SS1 and SS2 in patients with acute STEMI. Our aim in the present study was, therefore, to assess the relationship between these enzymes and SS1 and SS2 in patients suffering from acute STEMI.

## Methods

Our prospective study recruited 102 consecutive patients (24 female and 78 male; mean age=61±12.5 y) who had been admitted to our hospital with the diagnosis of acute STEMI and who underwent PCI between August 2018 and December 2018. The definition of acute STEMI was the presence of the pertinent criteria as follows: the detection of a rise and/or a fall in cardiac troponins with at least 1 value above the 99th percentile of the upper reference limit and with at least one of the following features: ischemia-related symptoms; new or presumably new ST-segment elevations in ≥2 contiguous leads with the cutoff point ≥0.2 mV in the anterior leads or new left bundle branch blocks; the development of pathological Q waves in the electrocardiogram; imaging evidence of new loss of viable myocardium or new regional wall motion abnormalities; and the identification of an intracoronary thrombus by angiography.^25^ Each one of the participating patients had type 1 MI.^25^

According to the study protocol, the exclusion criteria were defined as patients with a history of recent MI; those who had received any thrombolytic agent as a pretreatment; those with active infection or chronic inflammatory diseases; those with severe hepatic, renal, or hematological disease; and those with any history of neoplasm.

All the patients were evaluated with a detailed medical history and a thorough physical examination, recording such baseline demographic features as age, sex, hypertension, chronic obstructive pulmonary disease, peripheral arterial disease, smoking habits, diabetes mellitus, and CAD.

All the participating patients provided a written or oral-witnessed informed consent at the emergency service. Our study was performed in accordance with the principles of the Declaration of Helsinki and was approved by the institutional ethics committee.

All the transthoracic echocardiographic assessments of the study participants were performed using the Vivid S5 (GE Vingmed Ultrasound AS, Horten, Norway). The left ventricular ejection fraction was calculated using the modified Simpson rule. All the conventional echocardiographic examinations were performed according to the standards of the American Society of Echocardiography.^26^

All the patients were treated in accordance with the recommendations of the acute STEMI guideline.^27^ Once the written informed consent for cardiac catheterization was obtained, an emergency coronary angiography was performed in all the patients using the standard techniques. A glycoprotein IIb/IIIa inhibitor (tirofiban) was administered to the patients in the catheterization laboratory at the operator’s discretion. The decision regarding the implementation of PCI, coronary artery bypass graft surgery, or medical treatment was given by a heart team comprising 2 cardiologists and 1 cardiovascular surgeon. All the PCI procedures were performed using the standard clinical practice, and the choice between the alternatives of drug-eluting stents and bare-metal stents was at the operator’s discretion. The stenting of infarct-related arteries was successfully fulfilled in all the patients.

Blood samples were obtained from every eligible patient through venipuncture on admission to the emergency department. The samples were then immediately transferred to the laboratory. The blood was kept for 30 minutes at room temperature (22 ^°^C) to activate the clotting process, followed by centrifugation at 3000 rpm for 10 minutes. The ultimate serum samples were stored at −80 ^°^C until the time of the analyses of serum PON-1 and ARE activities. Serum PON-1 and ARES activities were measured using spectrophotometric commercial kits (Relassay, Gaziantep, Turkey) in the Cobas C 501 AutoAnalyzer (Roche Diagnostics, Mannheim, Germany). 

Routine serum biochemical parameters were measured using standard laboratory methods. All the patients needed to be fasting for at least 12 hours before antecubital blood was collected. The blood was placed for 30 minutes at room temperature (22 ^°^C) to activate the clotting process, followed by centrifugation at 3000 rpm for 10 minutes. The serum samples were aliquoted and stored at −80 ^°^C until the time of the analyses of routine serum biochemical parameters (Cobas C 8000 AutoAnalyzer, Roche Diagnostics, Mannheim, Germany).

PON-1 activity was determined using paraoxon as a substrate in the presence of sodium chloride (salt stimulated activity). The rate of paraoxon hydrolysis (diethyl-p-nitrophenyl phosphate) was measured by monitoring the increase in absorbance at 412 nm and 37 ^°^C by adding 20 μL of the stored serum to 200 μL of the Tris buffer containing 2 Mm of CaCI_2 _and 7 Mm of paraoxon.^11^^, ^^28^ PON-1 activity was expressed as U/L.

ARE enzyme activity was measured by using phenyl acetate as a substrate. The stored serum was added into the substrate, and the increase was read in the absorbance degree at 270 nm. The activity of the enzyme was extrapolated from the molar absorptivity coefficient of the resultant phenol, 1310 M^-1 ^cm^-1^. One unit of ARE activity, which is expressed as kU/L, was defined as 1 µmol of phenol generated in 1 minute under the assay’s conditions.^29^

**Figure 1:**
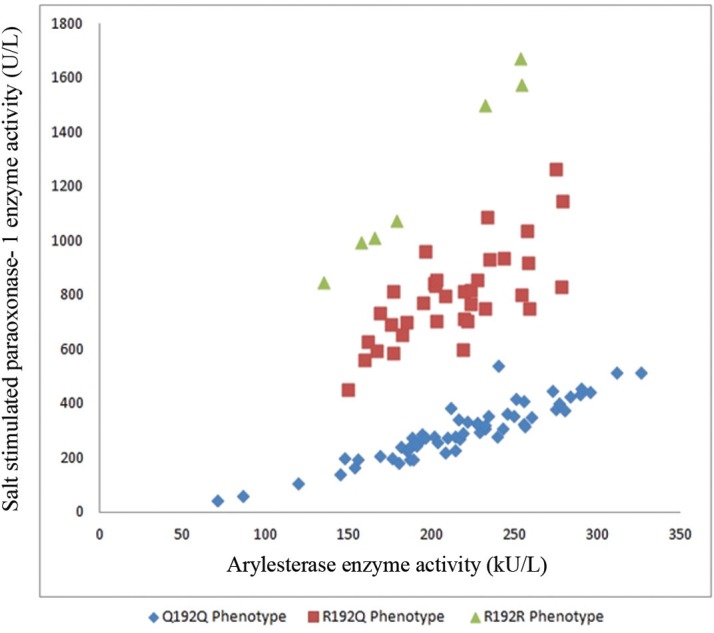
Paraoksonase-1 phenotype distribution of the patients with acute ST-segment elevation myocardial infarction (STEMI)

**Figure 2:**
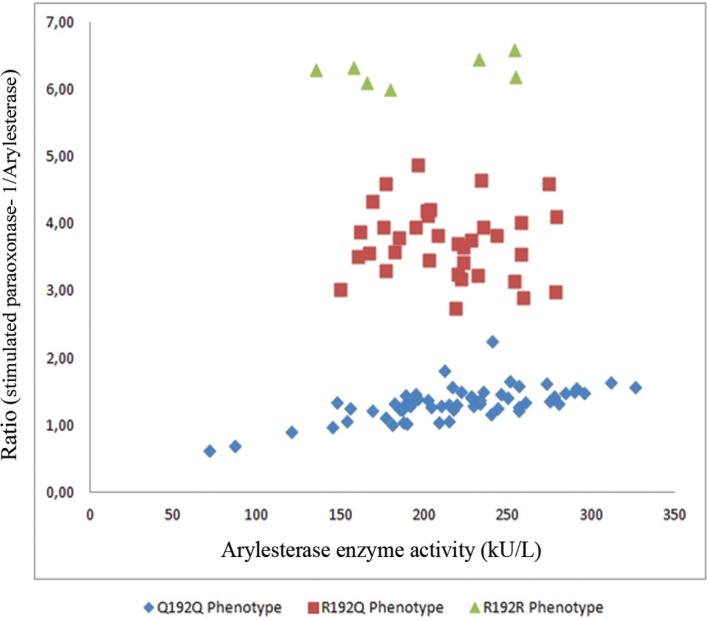
Ratio of paraoxonase-1 to arylesterase enzyme activities, corresponding to the QR phenotypic distribution of paraoxonase-1 enzyme activity

The amino acid substitution at codon 192 of the enzyme molecule (glutamine [Q]/arginine [R]) is one of the 2 major polymorphisms that PON-1 displays, which was suggested to show a close correlation with the enzymatic activities of PON-1 against several substrates including paraoxon and lipid peroxides.^14^^, ^^19^^, ^^30^^-^^36^ On the basis of this close correlation, we did not make a true genotyping such as polymerase chain reaction, but rather we extrapolated the corresponding 192_QR_ genotypes from the phenotyping method of PON-1 enzyme activity according to the ratio of salt-stimulated PON-1 to ARE. PON-1 activity was demonstrated to fit into one of the 3 phenotypes on the basis of the ratio of the salt-stimulated PON-1 activity to ARE activity (PON-1/ARE) as follows: homozygous low activity (Q192Q), heterozygous activity (Q192R), and homozygous high activity (R192R). Moreover, the respective PON-1/ARE ratios on the basis of which PON-1 phenotypes are recognized are ≤2.5 for Q192Q, 2.5–5.5 for R192Q, and ≥5.5 for R192R (Figure 1 and Figure 2).^32^^, ^^33^ This genotypic extrapolation method from the phenotypic high, intermediate, and low PON-1 activities on the basis of the PON-1/ARE ratio is compatible with the previous studies.^14^^, ^^19^^, ^^30^^-^^36^

The assessment of the cineangiographic views was performed using Axiom (Siemens Medical Solution, Erlangen, Germany) workstation by 2 experienced cardiologists, blinded to the study data. Each lesion with a diameter stenosis ≥50% in coronary vessels ≥1.5 mm in diameter was scored using the online SYNTAX Score calculator (http://www.syntaxscore.com). If the cardiologists were conflicted about the lesions, the ultimate score was decided by averaging the scores calculated by each cardiologist. The SS1 and SS2 scores were obtained for each patient. The patients were thereafter allocated further into 3 subgroups according to the SS1 categories (Group 1: low lesion complexity, SS1<23; Group 2: moderate lesion complexity, SS1 23–32; and, Group 3: high lesion complexity, SS1 ≥33). The SS2 scores, on the other hand, were given to predict 4-year mortality for the patients undergoing myocardial revascularization by way of PCI^18^ and were not stratified in the same manner as in the SS1 scores.

The statistical analyses of the study data were performed using SPSS software, version 21.0, for Windows (IBM SPSS Statistics for Windows, Version 21.0. Armonk, NY: IBM Corp., USA). The variables were tested for normality using the Kolmogorov–Smirnov and Shapiro–Wilk tests. The continuous variables were expressed as the mean±standard deviation, while the categorical variables were expressed as numbers and percentages. The independent *t*-test and the one-way ANOVA test were utilized in order to compare the differences between the groups. Further, the Duncan multiple comparison test was used to mark the means between which ANOVA showed significant differences. The Pearson χ^2 ^test was employed to analyze the relationship between the categorical variables. The correlations between the continuous variables were shown using the Pearson correlation coefficient. A further multiple linear regression analysis was carried out to obtain a predictive model for SS1 and SS2. A P value <0.05 was accepted to be statistically significant for all the statistical analyses.

## Results

A total of 102 eligible patients were included in the study. Seventy-eight patients were male, while 24 were female. The mean age of the patients was 61.14±12.25 years. The baseline clinical and demographic characteristics of the patients are depicted in Table 1.

**Table 1:** Demographic and clinical characteristics of the study patients*

Variable	Value
Female/Male	24 (23.5) /78 (76.5)
Age (y)	61.14±12.25
Glucose (mg/dL)	131.32±69.73
GFR (mL/min/1.73 m^2^)	80.51±21.57
TG (mg/dL)	172.58±91.54
T-Chol (mg/dL)	169.87±45.00
LDL-C (mg/dL)	94.68±39.91
HDL-C (mg/dL)	41.68±11.00
WBC (×10^9^/L)	9.79±3.62
Hb (g/dL)	13.80±1.94
Plt (×10^9^/L)	262.90±92.76
Neutrophil (×10^9^/L)	6.57±3.41
MPV (fL)	0.82±10.36
CRP (mg/dL)	1.70±3.21
Syntax I score	19.82±9.73
Syntax II score	32.36±11.53
PON-1 activity (U/L)	536.41±37.86
ARE activity (kU/L)	214.53±45.62
LVEF	43.51±5.91
Diabetes mellitus	32 (31.4)
Coronary artery disease	14 (13.7)
Hypertension	45 (44.1)
Peripheral arterial disease	1 (1.0)
Hyperlipidemia	24 (23.5)
COPD	7 (6.8)
Smoking	57 (55.9)

GFR, Glomerular filtration rate; TG, Triglyceride; T-Chol, Total cholesterol; LDL-C, Low-density lipoprotein cholesterol; HDL-C, High-density lipoprotein cholesterol; WBC, White blood cell; Hb, Hemoglobin; Plt, Platelet; MPV, Mean platelet volume; CRP, C-reactive protein; PON-1, Paraoxonase-1, ARE, Arylesterase; LVEF, Left ventricular ejection fraction; COPD, Chronic obstructive pulmonary disease

* Values are given as mean±SD or n (%).

The mean SS1 and the mean SS2 of the patients were 19.82±9.73 and 32.36±11.53, respectively. Thirty-two (31.4%) patients had a history of type 2 diabetes mellitus, 14 (13.7%) CAD, 45 (44.1%) essential hypertension, 1 (1%) peripheral arterial disease, and 7 (6.8%) chronic obstructive pulmonary disease. Further, 57 (55.9%) patients had smoking habits.

There was a statistically significant correlation between SS1 and SS2 (r=0.669, P<0.001). According to the SS1 scores, the distribution of the patients was as follows: low SS1, 57 (55.8%) patients; moderate SS1, 35 (34.3%) patients; and, high SS1, 10 (9.8%) patients. As for the distribution of the patients according to the PON-1 phenotype, 60 (58.8%) patients were found to be allocated to the Q192Q, 35 (34.3%) to the R192Q, and 7 (6.8%) to the R192R phenotypes (Table 2, Figure 1, and Figure 2). With respect to the phenotypic distribution of the patients among the low, moderate, and high SS1 scores, the distribution was observed to be homogeneous (χ^2^= 4.895,P>0.05) (Table 2).

**Table 2:** Phenotypic distribution of the patients according to the SYNTAX I Score category

	SYNTAX I Score Category			Total Number
	Low	Intermediate	High	
Phenotype				
Q192Q	36	20	4	60
R192Q	18	11	6	35
R192R	3	4	0	7
Total number	57	35	10	102

(χ^2 ^= 4.895,P=0.298)

Values are given as numbers.

When SS1 and SS2 were compared pair-wise between the 3 Q192R genotypic subgroups by use of ANOVA, a homogenous distribution was observed with regard to SS1 and SS2 between the genotypic subgroups (P=0.713 and P=0.253, respectively) (Table 3).

Six (5.8%) patients were admitted to the emergency department with an accompanying cardiogenic shock (CS). When SS1 and SS2, as well as serum PON-1 and ARE activities, were compared between the patients with CS and without CS, the SS1 and the SS2 scores were calculated to be significantly greater in the patients with CS (P=0.044 and P<0.001, respectively). Neither PON-1 activities nor ARE activities, however, were revealed to be significantly different upon comparison between the CS patients and non-CS patients with acute STEMI (Table 4).

When PON-1 and ARE activities and the SS2 scores were compared between the lower, intermediate, and higher SS1 categories, PON-1 and ARE activities did not show any significant difference. However, SS2 was revealed to show a significant relationship with SS1 (Table 5).

Table 6 displays the localizations of acute STEMI in the study population. The ANOVA revealed that PON-1 and ARE activities were not different between the different acute STEMI localizations. The SS1 and SS2 scores, on the other hand, were significantly different between the different localizations with the highest SS1 and SS2 in the anterior localization and with the respective lowest scores in the inferior and anterolateral localizations.

A multiple linear regression analysis with the stepwise method was implemented in an attempt to obtain a prediction model for SS1 and SS2. Although the analysis did not yield a model with a robust prediction capability for SS1, a model with a high predictive strength was achieved for SS2. According to this model, the glomerular filtration rate, gender, age, the presence of CS, and acute STEMI localization were able to predict SS2 with 87.5% precision (Table 7).

**Table 3:** Comparison of the different Q192R phenotype-corresponding subgroups on the basis of PON-1/ARE activity ratios and the SYNTAX I and SYNTAX II Scores*

	Q192Q Phenotype (n=60)	R192Q Phenotype (n=35)	R192R Phenotype (n=7)	P (overall)	P*	P**	P***
SYNTAX I Score	19.22±8.91	20.42±11.05	21.92±10.74	0.713	-	-	-
SYNTAX II Score	30.91±11.88	34.96±11.47	31.85±5.55	0.253	-	-	-
PON-1 activity (U/L)	300.78±84.48	799.05±69.77	1242.85±32.66	<0.001	<0.001	<0.001	<0.001
ARE activity (kU/L)	217.08±50.42	213.65±35.83	197.04±49.19	0.547	-	-	-

PON-1, Paraoxonase-1; ARE, Arylesterase;

P^*^, P value for the comparison of the Q192Q and R192Q groups

P^**^, P value for the comparison of the Q192Q and R192R groups

P^***^, P value for the comparison of the R192Q and R192R groups

* Values are given as mean±SD.

**Table 4:** Comparison of the SYNTAX Scores and PON-1 and ARE activities between the patients with or without accompanying CS on admission*

	CS Absent on Admission (n=96)	CS Present on Admission (n=6)	P value
SYNTAX I Score	19.40±9.75	28.00±5.56	0.044
SYNTAX II score	31.49±10.83	49.32±12.17	0.001
PON-1 activity (U/L)	538.44±43.20	497±29.66	0.791
ARE activity (kU/L)	214.07±45.40	223.42±55.79	0.658

CS, Cardiogenic shock; PON-1, Paraoxonase-1; ARE, Arylesterase

* Values are given as mean±SD.

**Table 5:** Association between the categorized SYNTAX I Score groups and SYNTAX II Scores and PON-1 and ARE activities*

Categorized SYNTAX I Scores	SYNTAX II Score	PON-1 Activity (U/L)	ARE Activity (kU/L)
Low (score<23)	26.74±8.18	514.85±29.34	216.82±36.72
Intermediate (score 23-32)	37.20±9.05	527.60±56.31	203.95±55.97
High (score ≥33)	47.48±14.60	690.10±11.07	238.48±45.65
P value	<0.001	0.316	0.091

PON-1, Paraoxonase-1; ARE, Arylesterase

* Values are given as mean±SD.

**Table 6:** Association between the localizations of acute STEMI and the SYNTAX I and SYNTAX II Scores and PON-1 and ARE activities*

Localization of Acute STEMI	SYNTAX I Score**	SYNTAX II Score***	PON-1 Activity (U/L)	ARE Activity (kU/L)
Anterior (n=54)	23.62±7.65	36.58±10.77	547.57±32.71	215.73±50.82
Inferior (n=40)	14.85±10.29	27.07±10.23	538.40±64.86	210.84±39.31
Lateral (n=2)	15.00±0.02	36.25±13.93	325.00±34.35	232.84±25.92
Anterolateral (n=3)	19.66±10.53	26.86±8.79	338.00±27.01	222.00±65.95
Inferoposterior (n=3)	21.00±12.52	29.90±16.60	648.33±88.05	222.40±35.54
P value	<0.001	0.001	0.705	0.943

PON-1, Paraoxonase-1; ARE, Arylesterase; STEMI, ST-elevation myocardial infarction

* Values are given as mean±SD.

** P<0.001 for Anterior vs. Inferior; P=0.184 for Anterior vs. Lateral; P=0.457 for Anterior vs. Anterolateral; P=0.622 for Anterior vs. Inferoposterior; P=0.982 for Inferior vs. Lateral; P=0.371 for Inferior vs. Anterolateral; P=0.254 for Inferior vs. Inferoposterior; P=0.569 for Lateral vs. Anterolateral; P=0.982 Lateral vs. Inferoposterior; P=0.856 Anterolateral vs. Inferoposterior

*** P<0.001 for Anterior vs. Inferior; P=0.965 for Anterior vs. Lateral; P=0.001 for Anterior vs. Anterolateral; P=0.295 for Anterior vs. Inferoposterior; P=0.240 for Inferior vs. Lateral; P=0.974 for Inferior vs. Anterolateral; P=0.660 for Inferior vs. Inferoposterior; P=0.340 for Lateral vs. Anterolateral; P=0.518 Lateral vs. Inferoposterior; P=0.730 Anterolateral vs Inferoposterior

**Table 7:** Multiple linear regression analysis for the SYNTAX II Score

Variable	β	SE	P value
GFR	−0.237	0.033	<0.001
Gender	6.568	1.427	<0.001
Age	0.322	0.058	<0.001
CS on admission	13.394	2.879	<0.001
Localization of acute MI	−2.008	0.556	0.001

β, Regression coefficients; SE, Standard error; R^2^=0.875

GFR, Glomerular filtration rate; CS, Cardiogenic shock; MI, Myocardial infarction

## Discussion

The main findings of our study can be summarized as follows: 1) serum PON-1 and ARE activities did not significantly change through low, intermediate, and high SS1 categories in the acute STEMI patients; 2) the SS2 scores also did not show any significant correlation with both PON-1 and ARE activities; 3) most of the patients with acute STEMI were allocated into the Q192Q phenotype (homozygous high risk), despite a homogenous distribution of the percentage of the patients with the respective SS1 and SS2 scores between the 3 phenotypic groups; 4) despite having greater mean SS1 and SS2 scores, the acute STEMI patients with CS on admission did not have any significantly high serum PON-1 and ARE activities, compared with those without CS on admission; and 5) the activity of neither serum PON-1 nor ARE possessed a significant relationship with the localization of acute STEMI. Accordingly, the fact that we utilized both SS1 and SS2 in the comparison of the enzyme activities conferred an additional strength to our study. To our knowledge, this is the first study to evaluate comprehensively the relationship between serum PON-1 and ARE activities and both SS1 and SS2 in patients with acute STEMI.

Evidence suggests that PON-1 and ARE activities are associated with the presence and extent of the disease in patients with stable CAD. ^11^^, ^^12^^, ^^19^ Akçay et al.^   37 ^ reported increased PON-1 activity in patients with metabolic syndrome compared with healthy controls. They also demonstrated a negative correlation with the Gensini score in metabolic syndrome patients with stable CAD, despite the absence of a significant difference regarding PON-1 activities between the metabolic syndrome patients with and without stable CAD. Gur et al.^   19 ^ also demonstrated high PON-1 and ARE activities in stable CAD patients compared with healthy controls. Aside from these premises, however, these enzyme activities did not display the same correlation with both the presence and the extent of CAD in the state of acute MI. In this regard, Erenler et al.^        38 ^ showed no significant difference in PON-1 activity upon making a comparison between acute STEMI patients and those with acute NSTEMI, despite the presence of significantly low activities of PON-1 compared with healthy controls. Moreover, they also could not reveal a significant correlation between the extent of CAD assessed by the Gensini score and the activity of PON-1 in patients with acute MI. In another study by Aksoy et al.,^        23 ^ conducted on 42 patients with acute MI (17 acute STEMI and 25 acute NSTEMI), PON-1 and ARE activities were demonstrated not to be associated with the SS1 score subgroups, despite greater oxidative stress indices accompanying the greater SS1 scores. In our study, we also did not find any correlation between PON-1 and ARE activities and both SS1 and SS2. In contrast to our study, where we inquired the relevant enzyme activities throughout the 3 SS1 categories, they only divided the patients into 2 categories as low SS1 (score <22) and high SS1 (score >22). We noted even further that PON-1 and ARE activities displayed no significant change not only among the 3 SS1 categories but also among the patients with differing SS2 scores.

As for the PON-1 Q192R polymorphism, current literature has yielded conflicting results regarding the association between the PON-1 polymorphism and CAD. While some studies have revealed results in favor of such an association,^39^^, ^^40^ some others have opposed this relationship.^41^^, ^^42^ Tang et al.^        43 ^ reported in their prospective study conducted on 3668 stable patients that ARE activity exerted more crucial atheroprotective function than PON-1 activity and anticipated better the 3-year major adverse cardiovascular events. Of note, they also mentioned the ineffectiveness of the genetic polymorphism of PON-1 in the prediction of major adverse cardiovascular events. Similarly, Birjmohun et al.^        44 ^ reported in their 6-year prospective study including 1138 apparently healthy individuals that the PON-1 Q192R polymorphism did not anticipate future fatal or nonfatal CAD. They rested their findings upon the premise that it was not only PON-1 activity itself but also the other HDL particle-related properties that concomitantly defined the causality between PON-1 and atherogenesis. Furthermore, Herrmann et al.^   41 ^ assessed a total of 642 patients with acute MI in terms of a probable effect of PON-1 Q192R genotypes in the occurrence of acute MI compared with controls, and found that the PON-1 Q192R polymorphism was not associated with acute MI and the severity of CAD. Nearly 50% of the patients in their study possessed the Q192 genotype, and only around 10% had the R192R genotype. Such a genotypic distribution is very close to that of our study, although we enrolled a relatively smaller patient group. Considering the above findings as a whole, it will be prudent to assume that PON-1 and ARE enzyme activities are associated neither with SS1 and SS2 nor with the genetic polymorphism of PON-1. Our study findings also support this.

Our study should be evaluated in light of some limitations. Our study population is relatively small. The majority of the patients were male and relatively older aged. The distribution of the clinical risk factors of CAD is not homogeneous. We did not enroll a control group, and nor did we interrogate the ethnic variation of the study population. Previous history for drugs used, especially history for lipid-lowering drugs, was not sought in the study. We also did not follow the patients and did not correlate our findings with future major adverse cardiovascular events, which may serve as a protocol for further studies.

## Conclusion

Our study showed that admission serum PON-1 and ARE activities were not associated with SS1 and SS2 and the localization of acute MI. Moreover, the 3 PON-1 Q192R genetic functional polymorphism groups displayed a homogenous distribution regarding the SS1 and SS2 scores in our patients with acute STEMI. Future larger scale studies are warranted to justify our findings.

## Notes:

***This paper should be cited as: ***Sökmen E, Çelik M, Sivri S, Güçlü K. Relationship between Paraoxonase-1 and Arylesterase Enzyme Activities and SYNTAX I and II Scores in Patients with ST-Elevation Myocardial Infarction. J Teh Univ Heart Ctr 2019;14(4):156-164.
